# Household Cooking Frequency of Dinner Among Non-Hispanic Black Adults is Associated with Income and Employment, Perceived Diet Quality and Varied Objective Diet Quality, HEI (Healthy Eating Index): NHANES Analysis 2007–2010

**DOI:** 10.3390/nu11092057

**Published:** 2019-09-02

**Authors:** Nicole Farmer, Gwenyth R. Wallen, Li Yang, Kimberly R. Middleton, Narjis Kazmi, Tiffany M. Powell-Wiley

**Affiliations:** 1 National Institute of Health Clinical Center; Bethesda, MD 20811, USA; 2 National Heart, Lung and Blood Institute; Bethesda, MD 20811, USA; 3 National Institute on Minority and Health Disparities, Bethesda, MD 20811, USA

**Keywords:** NHANES, cooking, non-Hispanic Blacks, healthy eating index, dietary behavior change, diet quality, perceived diet quality

## Abstract

Home cooking is associated with improved diet quality. Non-Hispanic Blacks, a population with diet-quality related health disparities, report lower home cooking than other racial/ethnic groups. Factors and subsequent dietary outcomes associated with this cooking disparity are relatively unknown. A secondary analysis was performed using demographic and consumer behavior data from the 2007–2010 cycles of the National Health and Nutrition Examination Survey (NHANES) to identify factors associated with household cooking frequency of dinner among Non-Hispanic Blacks. Self-reported dietary data were used to calculate Healthy Eating Index-2010 (HEI-2010) to determine cooking related objective diet quality. Lower income, unemployment, and higher perceived diet quality were significantly associated with higher cooking frequency (*p* < 0.05). For diet quality, higher vegetable (*p* = 0.031), lower empty calorie intake (*p* = 0.002), higher dinner time protein (*p* = 0.004) and lower dinner time dairy intake (*p* = 0.003) were associated with cooking. Total HEI scores were associated with higher cooking frequency for middle income (*p* = 0.007), but not higher or lower income categories (*p* = 0.306; *p* = 0.384), respectively. On average, factors associated with cooking frequency were psychosocial, income, and employment related. Objective diet quality as measured by HEI was variable. Future dietary studies among Non-Hispanic Blacks should include cooking, socioeconomic status and perceived diet quality as particularly relevant factors of interest.

## 1. Introduction

Poor diet quality is an important risk factor for chronic disease, including diet-related diseases such as hypertension, type 2 diabetes and cardiovascular disease [1]. Home cooking may improve diet quality by leading to a reduction in caloric intake, sodium intake, saturated fat, and an increase in fiber intake [2,3]. Based on population-based survey data, Non-Hispanic Blacks, a U.S. population with lower adherence to U.S. dietary guidelines [4] and higher prevalence of diet-related diseases [5], report lower cooking activity than other racial/ethnic groups. Cooking activity data from the U.S. Adult Time Use Survey showed Non-Hispanic Black women and men with the lowest reported cooking activity, and the only group with no significant recent increase in cooking activity when compared to Non-Hispanic Whites and Hispanic Americans [6]. An evaluation of cooking patterns using NHANES (National Health and Nutrition Examination Survey) data have found similar results, [7,8] in that Non-Hispanic Blacks were more likely to live in households with lower dinner cooking frequency when compared with Non-Hispanic Whites and Hispanic-Americans.

Demographic and socioeconomic status (SES) factors associated with higher cooking frequency among the general U.S. population include female gender, higher socioeconomic status (SES) as measured by income and education, increased age, increased household size, and classification of being food insecure [7,8,9]. Given the specific food culture and history among Non-Hispanic Blacks in the U.S., it is expected that factors affecting cooking frequency may be complex and multifactorial. However, specific attention to factors that may account for the lower cooking frequency among Non-Hispanic Blacks has not been fully explored outside of regionally based qualitative [10,11,12] and survey studies [13]. Focus groups among Non-Hispanic Black residents in North Carolina and Connecticut, which include lower income families from North Carolina, revealed a lack of cooking skills, as well as access to fresh produce as barriers to cooking and the use of fresh vegetables [10].

In terms of diet quality, dietary intake among Non-Hispanic Blacks for fiber, fruits and vegetables [14] is lower compared to other racial/ethnic groups. As well, Non-Hispanic Blacks have a larger percentage of calories obtained from added sugars [15]. Moreover, several longitudinal cohort studies show a role of diet quality impact on the risk of diet-related diseases, such as hypertension [5]. The differences in diet quality may occur from lower access to supermarkets in predominantly Non-Hispanic Black neighborhoods [16], culturally based food choices [12], or psychosocial factors, such as perceived diet quality [17]. Reports from the REGARDS (Reasons for Geographic and Racial Differences in Stroke) trial showed that the selection of foods from the Southern Dietary pattern was a primary mediator in hypertension risk for Non-Hispanic Black men and women [18]. The REGARDS study and other longitudinal studies exploring dietary patterns among Non-Hispanic Blacks have not reported dietary patterns by cooking/ meal preparation behaviors versus purchasing food behaviors. However, differing results from cohort studies evaluating the DASH (Dietary Approach to Stop Hypertension) diet may offer some insight into why taking cooking and meal preparation behaviors into account may be important. In both the DASH [19] and the DASH-sodium trial [20], in which foods for both the study and control arm were provided, blood pressure reduction was more for Non-Hispanic Blacks compared to Non-Hispanic White Americans. During the ENCORE (Exercise and Nutrition Interventions for Cardiovascular Health Study) trial, in which nutrition education but not food was provided to participants, Non-Hispanic Blacks had a smaller reduction in blood pressure and lower DASH scores compared to Non-Hispanic Whites [21]. These conflicting results could be explained by understanding food choice related to home cooking versus purchasing behaviors. Interestingly, Whitt-Glover, et al. showed significant improvement in vegetable consumption and diastolic blood pressure reduction through teaching cooking skills to Non-Hispanic Black adults as part of a DASH nutrition education intervention [22]. 

As there is a positive association between cooking frequency and higher diet quality for the general U.S. population, a reasonable question to consider is if some of the disparities in diet-related health conditions among Non-Hispanic Blacks may be the downstream manifestations of decreased cooking frequency. Secondarily, what is the expected diet quality of Non-Hispanic Blacks who cook frequently given neighborhood food access issues and perceived cultural food norms? Based on the deficiency in the current literature we sought to explore in a nationally representative sample: 1) If demographic and SES factors and perceived diet quality are associated with cooking frequency in a nationally representative sample of Non-Hispanic Blacks and 2) if cooking frequency is associated with dietary quality, using the Healthy Eating Index (HEI). Our first hypothesis was that SES factors would be significant limiting determinants of cooking frequency and could help to explain the reported cooking behavior disparities. Our second hypothesis was that cooking frequency among Non-Hispanic Blacks, who were not exposed to a cooking or nutrition intervention, would not be positively associated with dietary quality, consistent with the current literature. 

## 2. Materials and Methods 

### 2.1. Data and Sample Population Selection 

Data from the National Health and Nutrition Examination Survey (NHANES) were used for this analysis. NHANES is a cross-sectional survey designed to monitor the health and nutritional status of the civilian noninstitutionalized U.S. population [23]. Data are collected every 2 years. The NHANES sample is selected through a complex, multistage design that includes the selection of primary sampling units, households, and sample persons from selected households. The survey consists of interviews conducted in participants' homes, standardized physical examinations in mobile examination centers (MECs), and laboratory tests provided by participants during the physical examination [23]. Cooking frequency, our main variable of interest, was only fielded during the 2007–2008 and 2009–2010 cycles within the Consumer Behavior data section of NHANES. 

Non-Hispanic Blacks, Hispanics, persons with low income, and persons aged 60 and over were oversampled during 2007–2010 cycles [24]. Due to the NHANES complex sample design, sample weights and sample design variables were used to obtain unbiased estimates that are representative of the U.S. population [25,26]. Public-use data files from these two cycles of interest, 2007–08 and 2009–10, were combined in order to produce national estimates and standard errors [23,24,25]. For both 2007–08 and 2009–10 survey cycles, the provided two-year weights: WTINT2YR (all interviewed sample persons), WTMEC2YR (sample persons who have MEC data items) [4], and WTDRD1 (sampled person with dietary day 1 data) [27] were used to create new four-year weights following instructions provided by NHANES [24]. Preliminary statistical analyses were conducted in SAS Survey procedure (version 9.4, SAS Institute, Inc., Cary, NC, USA) using the masked variance units for strata (sdmvstra) and PSU/cluster (sdmvpsu) with corresponding weights [24]. 

Of the 20,686 participants aged 19 years or older who participated in the NHANES cycles 2007–2008 and 2009–2010, cooking frequency data were available for 20,375. Participants who self-identified as Non-Hispanic Black were selected from this number to yield a total of 2,336 participants for this analysis. Dietary data were available for 2242 participants. The dietary data were also analyzed to obtain data related specifically to dinner, discussed in more detail below. There were 2027 participants for these meal specific calculations.

### 2.2. Measures

#### 2.2.1. Cooking Related Variables 

The Flexible Consumer Behavior Survey (FCBS) module is a part of the Consumer Behavior section and provides personal interview data on dietary related consumer behavior topics [28]. Cooking frequency was evaluated using the NHANES consumer behavior question “In the past 7 days, how many times did you (or someone else in your household) cook dinner at home?” This particular question allows the respondent to answer at either an individual behavior level or household behavior level. As the maximum number of dinner meals for a week cannot exceed 7, responses that provided an answer > 7 were excluded. Responses were categorized in order to allow for practical interpretation and to maintain consistency with the current literature. Three categories were used for this analysis: 0–1 dinner cooked per week (‘low’), 2–5 (‘sometimes’) and 6–7 (‘high’) as previously used in Wolfson and Bleich [7]. Data were analyzed to ensure that these categories were robust for our sample population. 

The Consumer Behavior module also includes questions about an individual participant’s food choices, within the Diet Behavior and Nutrition (DBQ) questionnaire section [28]. The dichotomous question “Are you the person who does most of the planning or preparing of meals in your family?” was used to determine if adults in our sample were the main meal planner or preparer for their household. 

#### 2.2.2. Objective Diet Quality 

Dietary quality was determined from 24-hour dietary recall data collected from NHANES interviews. For both the 2007–08 and 2009–10 NHANES cycles, trained interviewers conducted the 24-hour dietary recalls in person at the mobile examination center using the U.S. Department of Agriculture Automated Multiple-Pass Methodology [29]. Day 1 dietary recall data were used because the majority of participants completed day 1 testing during the examination at the mobile examination center. Only dietary data determined reliable by interviewers were included in the NHANES database, and thus used in this analysis. 

Dietary recall data were used to calculate HEI, a diet quality index that measures conformance to federal dietary guidelines [30]. HEI was chosen as the measure of diet quality for this analysis because dietary adherence to US guidelines is reported as lower for Non-Hispanic Blacks [4]. Key features of HEI are that diet quality is assessed from the perspectives of adequacy and moderation; the scoring standards are density-based, such that the relative mix of foods is evaluated; and the standards for the maximum scores are the easiest to achieve recommendations among those that vary by energy level, sex, and/or age [30]. HEI-2010 was specifically chosen for this analysis because it corresponds with the data collection cycles and would thus reflect adherence to the dietary guidelines at the time of data collection. HEI-2010 is made up of 12 components that are summed to provide a total score with a maximum of 100 points. Nine of the 12 components (sub-scores) measure the consumption of adequate amounts of total fruit, whole fruit, total vegetables, greens and beans, whole grains, dairy, total protein foods, seafood and plant proteins, and fatty acids. The other three components measure moderate consumption of refined grains, sodium, and empty calories (solid fats, alcohol and added sugars—SoFAAS). For the moderate consumption components, lower numerical scores indicate higher consumption. Total scores for HEI range from 0 to 100 and higher scores indicate better diet quality. A score above 80 indicates good diet quality, a score of 51–80 indicates a need for improvement, and a score of 50 or below indicates a poor diet. Total nutrient intakes from 24-hour dietary data are used to determine daily HEI and component scores. 

The cooking frequency question used in NHANES is specific for dinner foods. To make a direct meal comparison of cooking frequency to diet quality, we determined dinner meal specific HEI-2010 total and component scores by selecting dinner foods and beverages from the 24-hour recall dietary records. Dinner foods were selected based on NHANES participants’ self-labeling of each food-eating occasion. Dinner related beverages were determined based on participants’ self-labeling of a beverage item in which consumption coincided with standard dinner hours (16:00–20:00) [31]. Both English and Spanish dinner and beverage self-labels were used. Summed total amounts for each participant’s dinner foods or beverages were calculated to determine total dinner nutrient amounts. 

The SAS program offered by NCI (National Cancer Institute) at https://epi.grants.cancer.gov/hei/sas-code.html was used to calculate HEI-2010 total and component scores for both total daily and dinner related intakes. All scores were analyzed as continuous variables. 

#### 2.2.3. Demographic and SES Co-variates 

Demographic and SES variables for this analysis were selected based on the current literature regarding determinants of cooking frequency for the general U.S. population [7,8,9]. Demographic and SES variables were defined using self-reported data [28]. Selected demographic variables were age, gender, marital status, and country of birth. SES variables selected were employment, income to poverty ratio, and education. All co-variates were collected during the NHANES interviewer-administrated questionnaires. 

After analysis of data frequencies among our sample population, some variables were recoded. After analysis of data frequencies, marital status was dichotomized into married or living with a partner, previously (divorced or widowed) or not married. Household size was changed to a dichotomous value of 1–3 persons or ≥4. Employment was determined using NHANES occupation variables from the Occupation Questionnaire Section, with a primary focus on the participant’s work within the previous week [32]. These occupation variables were chosen to correspond to the cooking frequency question time frame. Work status was calculated using NHANES occupation variables ‘type of work done last week’ and ‘hours worked last week at all jobs’. Variables were recoded as presented in Wolfson and Bleich [7] to create employment status (non-employed - including the unemployed, retirees, students, and those not actively looking for work), part time (1–34 h/week), full time (≥ 35 h/week). Income to poverty ratio is the ratio of income to the federal poverty threshold based on family composition and size. It is a valid indicator of SES and is used in NHANES data analysis to compare across survey years as the income thresholds are adjusted for inflation [33]. This parameter was used to develop cut-offs for three income subgroups: lowest (≤ 130% of the poverty threshold), middle (131%–185%) and highest (> 185%) [17]. Education was calculated using NHANES demographic variables and was recoded into four categories: less than 9th grade, 9th–12th grade/HS GED, some college or Associates degree, college graduate or above. 

#### 2.2.4. Food Security and Food Assistance Co-variates

The Food Security section (FSQ) provides personal interview data on household food security, individual food security, Supplemental Nutrition Assistance Program (SNAP)/Food Stamp program benefits, and Women, Infants and Children (WIC) program benefits [34]. Food insecurity was determined using the NHANES calculated ‘Household food security category’ variable created from the US Food Security Survey Module (US FSSM) [35]. The selected household food security category (FSDHH) variable offers four response levels: Full food security, marginal food security, low food security, very low food security. The variable used for food security was created based on affirmative responses to create the following four response categories: 1 = Adult full food security: no affirmative response in any of these items; 2 = Adult marginal food security: 1–2 affirmative responses; 3 = Adult low food security: 3–5 affirmative responses; 4 = Adult very low food security: 6–10 affirmative responses. Receipt of food stamp assistance and or SNAP (Supplemental Nutrition Assistance Program) benefits and WIC were determined using the NHANES question for assessing if benefits were received within the 12 months before the questionnaire administration. 

#### 2.2.5. Perceived Diet Quality (PDQ) Co-Variate 

In addition to demographic and SES factors, psychosocial factors, such as the perception of diet, may be important contributors to dietary quality. Powell-Wiley, et al. [17] found that Non-Hispanic Blacks, along with Non-Hispanic Whites, had a positive association between perceived diet quality (PDQ) and objective diet quality. It is not known if perceived diet is associated with cooking frequency. For this analysis, PDQ, a measure of subjective diet quality, was determined using the NHANES Diet Behavior and Nutrition questionnaire, ‘In general, how healthy is your overall diet?’ on a 5-point Likert scale, with possible answers ranging from ‘excellent’ to ‘poor’. PDQ responses were recoded as ‘high’ for those who perceived their diet to be ‘excellent’ or ‘very good’, ‘medium’ for those who perceived their diet to be ‘good’ and ‘low for those who perceived their diet to be ‘fair’ or ‘poor’ [17]. 

### 2.3. Statistical Analysis

All data analyses were performed using SPSS Complex Samples IBM SPSS 24 (IBM Corp, Armonk, NY, USA) [36]. To test our first hypothesis, Chi-square tests were performed to evaluate the relationships between cooking frequency groups and categorical covariates. One-way Analysis of Variance (ANOVA) was used to assess the relationship between cooking frequency groups and the continuous demographic variable, age. Significant variables from bivariate analysis were entered into a multinomial logistic regression model to determine predictor variables of cooking frequency. Interrelationships between variables were assessed, and variables with significant correlations with others in the model were removed from the logistic regression model using a backward elimination regression method to obtain the final model. The ‘high cook’ group was used as the reference group in the model. 

For testing our second hypothesis, independent t-tests and one-way ANOVA were used to compare means of total HEI score among the study population across cooking frequency groups, categorical demographic variables, SES variables, and PDQ. HEI scores that were significantly associated with cooking frequency were entered into multiple linear regression models using each HEI score as the continuous variable outcome and cooking frequency as the exposure. Multiple linear regression models were adjusted for demographic variables, SES variables, PDQ and food security. A *p*-value less than 0.05 was considered significant for all analyses. 

## 3. Results

### 3.1. Sample Population Characteristics by Cooking Frequency 

Table 1 shows sample-weighted baseline characteristics for the 2007–2010 NHANES Non-Hispanic Black adult population across categories of cooking frequency groups. Approximately thirty-five percent of Non-Hispanic Black adults were from households where cooking dinner occurred 6–7 times per week (high cooked group). These adults were significantly more likely to be married or living with a partner, have more part time employment than full time employment, have a lower income to poverty ratio, low/very low food security, or reported a high PDQ. In contrast to those adults in the high cook group, the majority of Non-Hispanic Black adults (51%) came from households in which cooking dinner occurred medium (2–5 times per week), and these adults were significantly more likely to have full-time employment, higher income, marginal food security or reported a lower PDQ. 

### 3.2. Factors Associated with Cooking Frequency 

Table 2 shows odds ratios (OR) with calculated 95% confidence intervals (CI) for predictor variables of cooking frequency from multinomial regression analysis. After adjustment for co-variates, the factors that were positively associated with a Non-Hispanic Black adult being more likely to come from a household that low cooked were marriage and food security. Adults who were previously or not married were three times more likely to be in the low cook group than the high group (OR 3.06, CI 2.07–4.53). Adults who reported full food security were 2 times more likely to be in the low cook than the high cook group (OR 2.01, CI 1.29–3.15). 

Low PDQ was positively associated with an adult being more likely to come from a household that cooked medium versus high (OR 1.54, CI 1.14–2.09). The socio-demographic factors associated with likelihood of being in a ‘medium cook’ household compared to a ‘high cook’ household were age, employment and income. For each one-year increase in age, there was 1% lower odds of being in the medium cook group versus high (OR = 0.99, CI 0.98–0.99). Compared to adults from the highest income category, adults in the lowest income category had 34% lower odds (OR 0.66, CI 0.45–0.96) of being in the medium cook group compared to high. Adults who were not employed, had 37% lower odds of being in the medium cook group compared to the high cook group (OR = 0.63, CI 0.46–0.86).

### 3.3. Diet Quality by Cooking Frequency

Table 3 displays dinner and daily HEI-2010 diet quality results by cooking frequency. The mean total dinner HEI-2010 score for the sample population was 40.90 (SE = 0.35) but did not differ across cooking frequency groups. The mean total daily HEI-2010 score for the sample population was 46.58 (SE = 0.45) and did vary significantly across cooking frequency groups. There was no statistically significant relationship between calories (kcal) by cooking frequency group for either daily or dinner intake (*p* = 0.544 and *p* = 0.065, respectively). For both dinner and daily scores, adults in the high cook group were more likely to have higher vegetable intake (*p* = 0.03 dinner; *p* = 0.003 daily) and lower empty calorie intake (*p* = 0.008 dinner; *p* = 0.0001 daily) than those in the low or medium cook groups. For both the dinner and daily scores, adults in the medium cook group were more likely to have higher seafood and plant protein scores than adults in low or high group (*p* = 0.002 dinner; *p* = 0.001 daily). Adults in the high group were more likely to have higher dairy intake (*p* < 0.001), and total protein intake (*p* = 0.02) for dinner intake only. There were no significant differences across cooking frequency groups for HEI components sodium, refined grains, whole grains, fatty acid, greens and beans, and total and whole fruit. 

Table 4 and Table 5 show regression adjusted means for each HEI score significantly associated with cooking frequency. Models were adjusted for covariates related to each HEI score (e.g. gender, age, income, education), and mean differences represent beta-coefficients for each model. For total daily vegetable intake, adjusted mean scores varied across cooking frequency groups. Adults in the low cook group were likely to have a vegetable score 0.26 points less, out of a possible 5 points, than those in the high cook group (*p* = 0.031). However, total vegetable dinner scores did not vary across cooking frequency groups (*p* = 0.072 low to high; *p* = 0.818 medium to high). 

Both empty calorie intake and seafood intake, for dinner and daily adjusted means, were significant across cooking frequency groups. Daily adjusted means of empty calories were 1.19 points higher, equating to lower intake, among those who high cooked versus those who low cooked; and 1.42 points higher for dinner adjusted means (*p* = 0.002). Mean dinner time scores for protein intake were significantly higher by 0.42 of a point for those in the high cook group compared to the low cook group (*p* = 0.004). 

### 3.4. Income to Poverty Ratio Stratified Dietary Quality 

Supplementary Figure S1a–f show adjusted means for dietary quality by Total Dinner and Daily HEI scores for income categories: <130%, 131–185%, and > 185% income to poverty ratios. All models were adjusted for gender, marital status, education, food security, birthplace, PDQ, and age. The high cook group was the reference group for all models. Only adults with income between 131–185% had a significant different daily HEI score in the low cook versus high cook groups (*p* = 0.007). Although, there was a trend in the lower income group to have lower daily HEI in the medium group versus high cook group (*p* = 0.053), this failed to meet significance level. 

### 3.5. Meal-preparer Stratified Dietary Quality 

The meal preparer/planner status is an individual level question, and the cooking frequency question is at the level of the household. Therefore, there is no anticipated difference between meal preparers and non-meal preparers for our first research question, determination of factors associated with cooking frequency. We did, however, explore if meal preparer status stratification led to a difference in Total Daily and Dinner HEI score. We found that when the respondent was not the main preparer, then education (*p* = 0.013 daily; *p* = 0.001 for dinner), birthplace (*p* < 0.001 for daily and *p* = 0.001 for dinner), age (*p* < 0.001 for daily and *p* = 0.002 for dinner) and gender (*p* = 0.016 for dinner) were significant factors for total HEI score. If the respondent was the main preparer/planner, then age (*p* < 0.001), education (*p* = 0.003), birthplace (*p* < 0.001), PDQ (*p* < 0.001), and gender (*p* = 0.008) were also significant for Daily HEI score. However, Dinner HEI scores among main meal preparers/planners were not associated with gender (*p* = 0.079) nor PDQ (*p* = 0.095). Our results suggest it is important to determine the main preparer/planner role when evaluating the total daily HEI score. As well, that there is a differing role of gender between daily and dinner scores. 

### 3.6. Post-hoc Analysis: Gender and Marital Status 

Gender differences in cooking frequency are well reported in the literature [8,36]. We did not find a significant association between gender and cooking frequency. The cooking frequency question allowed for a response at either the individual or household level. Presuming a household level response, it is possible that men within the sample answered at the household level versus as the main meal planner/preparer within the home. We also considered that age may be a relevant individual level co-variate that may affect gender differences. To test this post-hoc hypothesis we examined the distribution of age and of meal planners/preparers in our sample by gender. Using Pearson Chi-square analysis, we found a significant gender difference for the variable main meal planner/preparer (*p* < 0.0001) as shown in Table 6. If men were not the main meal planner/preparer than this would suggest they were likely living with a partner or married. Our post-hoc analysis also found that men were significantly more likely to be married than women in our sample (*p* < 0.001). 

## 4. Discussion

Our cross-sectional analysis of NHANES data from 2007-2010 showed that among non-Hispanic Black adults not being married or having full food security was associated with less cooking frequency. However, having a lower income, unemployment, increased age or higher PDQ was associated with higher cooking frequency. We also found that higher cooking frequency was significantly associated with higher diet quality as measured for HEI components during daily and dinnertime intakes when controlling for SES variables, such as income and education. Consistent with prior reports of low cooking frequency among Non-Hispanic Blacks adults, we found most adults did not cook dinner at least 6 times per week [7,8]. These findings are in contrast to the current literature which reports that on average, a majority of American adults cook dinner at least 6 times per week. Dissimilar from the literature [8,9], we did not find that education was an independent factor associated with cooking frequency. Nor did we find a positive relationship between cooking frequency and the highest income level as found in Wolfson and Bleich [7] and Virudachalam, et al. [8]. 

There may be several explanations for our findings in contrast to the literature. One, in comparison to other analyses of cooking behavior and SES differentials, our findings could reflect differences concerning cooking behavior among Non-Hispanic Blacks, which may not be appreciated in mixed racial/ethnic analyses [37]. Two, the loss of a dichotomous relationship by income in our sample may actually represent choices not to cook that differ among SES groups within Non-Hispanic Black adults. For example, although higher income adults may have sufficient choices for food options outside of the home, increased participation in cooking may also be simultaneously influenced by the perception of foods that are culturally congruent or perception of time availability that may dissuade everyday home cooking [38,39]. Our finding suggests the likely importance of available time to cook in our sample in that not working was more likely to be independently associated with high cooking frequency. Furthermore, those adults in the medium cook group were more likely to have full-time employment. Three, cooking behavior choices reflect not only what is available based on SES situations, but also household level and individual attitudes related to ability, skills, and self-efficacy. For example, Garcia, et al. [13] in a cross-sectional survey of Non-Hispanic Black adults in Baltimore found self-efficacy for healthy foods was positively associated with cooking frequency. Our finding of a significant association of high PDQ and cooking frequency is consistent with the role of psychosocial factors and cooking behavior. Studies on the role of cooking and perceived diet quality of meal preparers show an inter-relationship between PDQ, and other psychosocial factors, such as self-efficacy and social support [40,41]. Unlike SES disparities in foods and nutrient intake, SES differences for cooking and food preparation behaviors have been less explored in the literature [42]. In summary, our results contribute to the understanding of overall dietary behaviors by suggesting that SES differentials for cooking among Non-Hispanic Blacks may not be the same as for other dietary behaviors, such as food purchasing behaviors [43]. Non-Hispanic Black men are reported to have the lowest cooking activity [6]. We did not find a significant gender difference by cooking frequency. This should be interpreted in the context of our finding that men were more likely to be married and less likely to be the meal planner/preparer. Thus, it is more likely that men answered the household cooking frequency question representing their counterpart’s cooking behavior. This supports the current literature [38,44]. Besides our finding of marriage being a significant independent factor related to cooking frequency and occurring at a higher frequency for men in our sample may showcase a cultural norm that Non-Hispanic Black women do more of the meal preparation at home than Non-Hispanic Black men. Although not exclusive to Non-Hispanic Blacks, this gender difference in the setting of other limitations to cooking (culture, or time availability as suggested indirectly by our data) may become compounded and contribute to Non-Hispanic Black men having greatest disparity in cooking. Of note, the role of marriage and cooking may vary across different Non-Hispanic Black populations as Garcia, et al. [13] found no significant role of marriage on home meal preparation for Non-Hispanic Blacks living in a Baltimore food desert. 

Higher cooking frequency is associated with low food security, presumably due to fewer options for food purchasing acquisition options [7], representing an interplay between what people have access to and what they know how to utilize. We also found similar results among Non-Hispanic Black adults, consistent with the literature [45,46]. We did not find that receipt of food assistance; specifically, SNAP which offers nutritional and cooking skills education, contributed to this association. However, the initiation of SNAP benefits with nutrition education promoting fruit and vegetable intake started in 2008 [47], before the midpoint of our analysis time frame. 

Given the burden of diet-related disease among Non-Hispanic Blacks in the U.S., it is important to identify and promote optimal dietary behaviors that may be associated with diet quality. By using HEI-2010 as our measure of objective diet quality, we were able to ascertain how the cooking frequency of Non-Hispanic Blacks can relate to adherence with recommendations of the Dietary Guidelines for Americans [30]. Promoting vegetable consumption has the potential to reduce CV risk by 20% [48]. However, Non-Hispanic Blacks are often found to have lower vegetable consumption than other racial/ethnic groups [4]. We found that Non-Hispanic Blacks from households that cook with a high frequency had favorable dietary behaviors of higher vegetable intake compared to those who cook at lower frequency. We also observed a lower intake of empty calories compared to those from households that cooked low or medium frequency. For the total daily HEI score, there was a difference across income levels for cooking frequency, with adults in the middle-income category having a significantly higher HEI score if they were from a household that high cooked versus low. Thus, the promotion of cooking may be an important dietary behavior, but just occurring at a lower frequency compared to other racial/ethnic U.S. groups to not have an overarching health impact. We also found a difference between vegetable intake for dinner and daily HEI scores. There is possibly a dietary behavior pattern associated with cooking that promotes more vegetable intake throughout the day, but not at dinner time. Or the dinner time consumption of vegetables may be impacted by SES variables present in the adjusted regression model for dinner, but not for daily vegetable intake. 

Through a secondary analysis of NHANES data on cardiometabolic mortality and dietary habits, Micha et al. showed that consumption of sugar sweetened beverages, a component of the HEI empty calorie score, was the highest dietary habit contributor to cardiometabolic mortality [49]. Our findings regarding lower consumption of empty calories and higher cooking frequency support exploring cooking as a behavior related to favorable nutritional choices associated with lower empty calorie intake. Foods classified as empty calories are not foods that necessarily require preparation or cooking for consumption, like vegetables. Thus, an association with lower consumption of empty calories and cooking suggests a healthier behavioral pattern of those adults who cook more. This is similar to findings from Wolfson and Bleich [7] that cooking frequency is associated with lower intake of fast food, and by that of Gustat, et al. [13] who found that Non-Hispanic Blacks living in New Orleans had lower consumption of purchased foods when home cooking was present at least daily. 

Our results are also consistent with those published by Tiwari, et al. [50] who found empty calorie consumption was associated with higher cooking among predominantly middle class obese Non-Hispanic White adults. The similar findings likely reflect an association with cooking that spans across cultures despite disparities in consumption of sugar sweetened beverage empty calories for Non-Hispanic Blacks [51,52]. 

Our findings also identify areas for discussion and future inquiry about cooking and diet quality among Non-Hispanic Blacks. For example, within the high cooking frequency group, the total HEI score remained below the reported U.S. average of 54.10 [30]. HEI total score was not associated with cooking frequency after adjusting for covariates. This may be explained by barriers to healthy eating among Non-Hispanic Blacks, which have been found to potentially decrease total HEI scores [39]. We also did not see an association with cooking frequency and sodium intake or refined grain intake. Higher amounts of total protein and lower amounts of dairy, both of which may be nutritionally adverse [53,54], were positively associated with cooking dinner in our sample. However, these associations only occurred in dinnertime preferences, suggesting an area of nutritional compensation during other meals of the day. Intake of seafood and plant protein was higher in the medium cook group than the low or high cook group and may represent choices to cook these types of foods or to purchase them. 

We should be cautious in interpreting some of our results. Since cooking frequency pertained to the last seven days and HEI based on 24-hour recall, it is possible that our results do not represent usual dietary and cooking behavior. In terms of our vegetable results, we did not find an increase in the intake of greens and beans. However, only of total vegetable score that may include consumption of processed vegetables or starchy vegetables like potatoes. Consumption of both vegetables and sugar sweetened beverages were significant components of the Southern Dietary pattern identified in the Jackson Heart Study [5]. Our results may differ from that of the Jackson Heart Study because the NHANES sample is not regionally based. Also, our sample was not stratified by health outcomes or diet-related disease status. Dietary quality and pattern reports from The Jackson Heart Study and other longitudinal studies, such as REGARDS [18], include participants that developed or who already had a diagnosis of a diet-related disease. Future studies that include health status stratification of cooking frequency among Non-Hispanic Blacks may help to provide more insight into the role of cooking behavior and diet-related disease disparities. 

## 5. Limitations

Our analysis has several limitations that should be considered. Our analysis is cross-sectional and thus cannot identify any causal relationships. Due to limitations with NHANES demographic variables, we are not able to assess the role of family structure, including the presence of children, which may influence cooking frequency. Nor could we determine the role of food access at the neighborhood level from our analysis. Our analysis is based on dietary self-report, and it is plausible that individuals from households with higher cooking frequency may have more recall of food intake compared to those from lower frequency cooking households. Food preparation behaviors are complex to define [42]. Cooking frequency was used as an indicator of behavior in this analysis. However, it remains unknown if similar results occur when other indicators such as cooking skills and knowledge, or cooking preparation time are used. NHANES dietary records reflect consumption of both home-cooked and food purchased outside of the home. Therefore, we are not able to comment specifically on the diet quality of home cooked food. Lastly, our analysis used the cooking frequency question from the 2007–2010 NHANES data cycles which were the only available data cycles for this variable. Although the current economic and social circumstances during the time of data collection may not reflect current times, current reporting in the literature of Non-Hispanic Blacks having lower cooking activity [6] suggests our analysis and conclusions may still be pertinent. 

## 6. Conclusions

National health policy agendas now put interventions to change eating patterns, along with physical activity patterns and tobacco use at the top of the list of strategies to address non-communicable diseases [55]. As public health and medicine move towards developing preventive approaches to diet-related illnesses, including tailored-interventions [56], it is essential to consider factors that promote positive dietary behaviors. Although our findings are cross-sectional, it may offer considerations for future research to address the cooking frequency disparity. Our findings suggest that possibly attention to factors such as income, food security and perceived diet quality, as well as the heterogeneity that may exist among Non-Hispanic Blacks, maybe important to consider. For instance, the promotion of higher cooking frequency among higher income Non-Hispanic Blacks may be related to promoting cooking as a viable option within their daily living schedules as opposed to focus on food access options [39]. Furthermore, regardless of cooking frequency, promoting the intake of vegetables at dinnertime requires attention to sociodemographic and socioeconomic factors which often parallel food access issues, which are the focus of ongoing public health and public policy research.

In summary, based on a national representative sample demographic, SES and psychosocial variables are associated with cooking behavior among Non-Hispanic Blacks, but in a dissimilar manner than reported in the literature for the general U.S. population. Further studies exploring cooking behavior among Non-Hispanic Blacks may gain insight by placing data in the context of factors independently associated with cooking behavior: Income, PDQ, marital status and employment. Diet quality related to cooking was variable in terms of HEI based intakes, but on average cooking of dinner at least 6–7 times per week was associated with higher adherence to dietary guidelines and more beneficial dietary quality as determined by vegetable intake and empty calorie intake regardless of SES and demographic factors. This analysis offers the first steps in exploring cooking behaviors among Non-Hispanic Blacks to better understand contributors to the cooking disparity reported in the literature. Lastly, for future studies to provide insights into the role of cooking behaviors in health outcomes among Non-Hispanic Blacks, ongoing inclusion of cooking and meal preparation behavior questions are needed in longitudinal and population-based studies. 

## Figures and Tables

**Table 1:** Characteristics for sample population of Non-Hispanic Black adults, age ≥ 19 years: NHANES, 2007–2010. (observations = 2336; weighted population 24,949,641).

	Total	Frequency of Cooking Dinner at Home per Week Mean, % US Population			*p*-value
		Low (0–1 time/week)	Medium (2–5 times/week)	High (6–7 times/week)	
Observations (*n*)		318	1147	871	
% Total U.S. population		13.60%	51.00%	35.50%	
Age (Mean, SE) Co-variate % (% within cooking frequency group)	43.80 (0.51)	42.56 (0.78)	42.9 (0.59)	45.95 (0.97)	**0.003**
Gender					
Male	44.8%	13.9% (45.9%)	50.5% (44.3%)	35.6% (44.8%)	0.841
Female	55.2%	13.3% (54.1%)	51.3% (55.7%)	35.4% (55.2%)	
Marital status- *n* = 2276					
Married or living with a partner	44.0%	8.0% (26.7%)	52.7% (45.3%)	39.2% (51.4%)	**<0.001**
Previously or low married	56.0%	17.4% (73.3%)	50.0% (54.7%)	32.6% (48.6%)	
Country of birth					
Born in U.S.	89.4%	14.2% (93.6%)	51.0% (89.3%)	34.8% (87.6%)	0.204
Not Born in U.S.	10.6%	8.1% (6.4%)	50.9% (10.7%)	41.0% (12.4%)	
Household Size					
1–3 people	61.5%	17.3% (78.6%)	50.3% (60.7%)	32.3% (56.1%)	**0.013**
≥ 4 people	38.5%	21.4% (21.4%)	39.3% (39.3%)	43.9% (43.9%)	
Employment					
Non-employed	44.1%	13.7% (44.8%)	44.2% (38.4%)	42.1% (52.6%)	**<0.001**
Part-time (1–34 hours/week)	13.9%	13.50% (13.9%)	50.7% (13.8%)	35.7% (14.0%)	
Full-time (≥ 35 hours/week)	42.0%	13.40% (41.4%)	58.3% (47.8%)	28.3% (33.4%)	
Income to poverty ratio % of poverty threshold					
lowest (< 130% )	32.1%	13.7% (33.0%)	45.2% (28.3%)	41.1% (37.4%)	**0.024**
middle (131%–185% )	16.9%	12.7% (16.2%)	49.1% (16.2%)	38.2% (18.3%)	
highest (> 185% )	51.0%	13.3% (50.9%)	55.9% (55.5%)	30.8% (44.4%)	
Education- *n* = 2278					
less than 9th grade	4.1%	10.1% (3.1%)	46.2% (3.7%)	43.7% (5.1%)	
9–12th grade	48.8%	13.5% (49.5%)	47.9% (45.5%)	38.6% (53.0%)	0.073
Some college or AA degree	32.0%	13.4% (32.3%)	56.6% (35.5%)	30.0% (27.2%)	
College graduate or above	15.1%	13.3% (15.1%)	52.0% (15.3%)	34.7% (14.7%)	
Adult food security-					
Full	63.6%	15.7% (73.5%)	50.7% (63.2%)	33.6% (60.1%)	**0.017**
Marginal	15.2%	8.9% (9.9%)	53.2% (15.9%)	37.9% (16.2%)	
Very Low/Low	21.3%	10.6% (16.6%)	49.9% (20.9%)	39.6% (23.7%)	
Household WIC benefits- n = 1635					
Yes	15.0%	7.4% (9.6%)	52.4% (14.9%)	40.2% (16.9%)	0.252
No	85.0%	12.3% (90.4%)	52.9% (85.1%)	34.8% (83.1%)	
Household food assistance or SNAP n = 881					
Yes	70.6%	11.8% (69.0%)	45.7% (66.6%)	42.5% (75.5%)	0.247
No	29.4%	12.6% (31.0%)	54.5% (33.4%)	32.9% (24.5%)	
Perceived Diet Quality (PDQ)					
Low	34.8%	17.1% (44.1%)	52.8% (36.2%)	30.1% (29.6%)	
Medium	40.1%	12.4% (36.7%)	52.1% (41.1%)	35.5% (40.3%)	**<0.001**
High	25.1%	10.5% (19.2%)	46.6% (22.7%)	43.0% (30.1%)	

Weighted percentages are presented for all data except age. Significance test using ANOVA done for age data. All other comparisons completed using Pearson Chi-Square. Bolded numbers indicate *p*-value <0.05. WIC Women Infant and Children; SNAP Supplemental Nutrition Assistance Program.

**Table 2:** Multinomial logistic regression model showing OR (odds ratio) for cooking frequency groups among Non-Hispanic Black adults (age ≥ 19 years): NHANES, 2007–2010. (observations = 2336; weighted population 24,949,641).

Predictor Variables	Frequency of Cooking Dinner at Home per Week Reference Group: High					
	Low (0–1 time/week)			Medium		
				(2–5 times/week)		
	**OR**	**95% CI**		**OR**	**95% CI**	
Age	0.99	0.98	1.00	**0.99**	**0.98**	**0.99**
Marital status *ref. group: married or living with a partner*						
Previously or low married	**3.06**	**2.07**	**4.53**	1.24	0.95	1.63
Employment *ref. group: full time (>34 hour/week)*						
Not working	0.70	0.45	1.10	**0.63**	**0.46**	**0.86**
Part time (1–34 hours/week)	0.68	0.39	1.21	0.71	0.46	1.10
Income to poverty ratio *ref. group: highest (>185% of poverty threshold)*						
lowest (<130% of poverty threshold)	0.73	0.47	1.13	**0.66**	**0.45**	**0.96**
middle (131%–185% of poverty threshold)	0.84	0.45	1.61	0.76	0.54	1.06
Food security *ref. group: low/very low*						
Full food security	**2.01**	**1.29**	**3.15**	1.06	0.75	1.49
Marginal food security	1.02	0.57	1.80	1.04	0.71	1.54
Perceived Diet Quality (PDQ) *ref. group: high*						
low	1.98	1.41	2.78	**1.54**	**1.14**	**2.09**
medium	1.27	0.96	1.67	0.76	0.54	1.067

Bolded numbers indicate *p*-value < 0.05. Age is a continuous variable. Reference category for cooking frequency is high (6–7 times/week). Co-variate reference group in *italics*.

**Table 3:** Mean comparison of diet quality measures for dinner and daily intake (kcal, total HEI score, and HEI components) by cooking frequency categories, among Non-Hispanic Black adults ≥ 19 years of age: NHANES, 2007–2010 (for dinner data observations = 2027; weighted population = 22,883,113; for daily data observations = 2242; weighted population = 25,392,351).

Frequency of Cooking Dinner at Home Each Week					
Total Energy (kcal)	Total Means (SE)	Low (0–1 time/week) Means (SE)	Medium (2–5 times/week) Means (SE)	High (6–7 times/week) Means (SE)	*p*-value
Dinner	824.20 (14.32)	802.74 (25.51)	826.54 (14.80)	852.89 (35.50)	0.544
Daily	2099.55 (38.16)	2022.15 (59.90)	2091.66 (37.13)	2293.88 (101.43)	0.065
HEI-2010 scores (maximum score)					
Total HEI score (100)					
Dinner	40.90 (0.35)	41.23 (0.65)	41.13 (0.51)	39.11 (0.84)	0.170
Daily	46.58 (0.45)	**43.96 (0.98)**	**46.76 (0.65)**	**47.20 (0.58)**	**0.023**
Total Vegetable (5)					
Dinner	2.94 (0.05)	**2.68 (0.14)**	**2.83 (0.76)**	**3.16 (0.11)**	**0.030**
Daily	2.62 (0.05)	**2.41 (0.69)**	**2.56 (0.06)**	**2.78 (0.08)**	**0.003**
Greens and Beans (5)					
Dinner	0.95 (0.06)	0.78 (0.12)	0.97 (0.72)	1.01 (0.12)	0.340
Daily	1.10 (0.07)	0.90 (0.09)	1.11 (0.09)	1.16 (0.13)	0.252
Total fruit (5)					
Dinner	0.75 (0.04)	0.74 (0.11)	0.77 (0.06)	0.73 (0.07)	0.890
Daily	2.06 (0.05)	1.84 (0.16)	2.09 (0.07)	2.09 (0.08)	0.360
Whole fruit (5)					
Dinner	0.38 (0.03)	0.34 (0.09)	0.41 (0.04)	0.37 (0.05)	0.740
Daily	1.67 (0.05)	1.49 (0.15)	1.69 (0.74)	1.68 (0.09)	0.493
Whole grains (10)					
Dinner	0.71 (0.07)	0.52 (0.13)	0.75 (0.09)	0.72 (0.11)	0.300
Daily	1.78 (0.06)	1.55 (0.19)	1.72 (0.09)	1.97 (0.12)	0.184
Total Dairy					
Dinner	2.58 (0.19)	**1.99 (0.17)**	**2.84 (0.17)**	**3.23 (0.94)**	**<0.001**
Daily	3.84 (0.17)	3.72(0.19)	3.81(0.13)	4.11(0.23)	**0.330**
Total protein (5)					
Dinner	4.14 (0.04)	**3.80 (0.94)**	**4.11 (0.05)**	**4.20 (0.06)**	**0.020**
Daily	4.39 (0.04)	4.36 (0.05)	4.42 (0.03)	4.11 (0.23)	0.330
Seafood and plant protein (5)					
Dinner	0.84 (0.05)	**0.55 (0.09)**	**0.98 (0.07)**	**0.76 (0.09)**	**0.002**
Daily	1.62 (0.05)	**1.37 (0.09)**	**1.77 (0.07)**	**1.51 (0.08)**	**0.001**
Fatty Acid (10)					
Dinner	5.61 (0.10)	5.33 (0.35)	5.58 (0.19)	5.74 (0.16)	0.620
Daily	5.54 (0.09)	4.86 (0.26)	5.62 (0.13)	5.44 (0.15)	0.063
Sodium (10) ^					
Dinner	3.58 (0.13)	3.89 (0.25)	3.62 (0.17)	3.41 (0.19)	0.400
Daily	4.72 (0.11)	4.94 (0.24)	4.71 (0.14)	4.63 (0.20)	0.690
Refined grains (10) ^					
Dinner	5.99 (0.10)	5.83 (0.31)	5.97 (0.16)	6.09 (0.19)	0.830
Daily	6.51 (0.07)	6.52 (0.28)	6.53 (0.09)	6.47 (0.15)	0.966
Empty calories (SoFAAS) (20) ^					
Dinner	12.43 (0.18)	**11.34 (0.35)**	**12.24 (0.22)**	**12.99 (0.32)**	**0.008**
Daily	10.81 (0.19)	**9.68 (0.37)**	**10.67 (0.25)**	**11.37 (0.23)**	**0.001**

Significance test using ANOVA. Bolded numbers indicate *p*-value < 0.05. ^ Higher score equates to lower consumption. SoFAAS- Solid fats, alcohols, and added sugars, referred to as 'empty calories'.

**Table 4:** Adjusted means from multiple linear regression models of daily HEI total score and components and cooking frequency groups among Non-Hispanic Black adults ≥ 19 years of age: NHANES 2007–2010 (observations = 2242; weighted population = 25,392,351).

	Total Daily HEI Score ^a^ (Max Score 100)		Total Daily Vegetable ^b^ (Max Score 5)		Daily Empty Calorie ^c^ (Max Score 20)		Daily Seafood and Plant Protein ^d^ (Max Score 5)	
	Adjusted Mean (SE)	*p* value	Adjusted Mean	*p* value	Adjusted Mean	*p* Value	Adjusted Mean	*p* value
Low	47.98 (1.36)	0.057	**2.43 (0.11)**	**0.031**	**11.74 (0.48)**	**0.002**	1.75 (0.16)	0.913
Medium	49.79 (0.85)	0.532	2.54 (0.07)	0.158	12.42 (0.39)	0.100	**1.99 (0.11)**	**0.046**
High (reference group)	50.23 (0.90)		2.69 (0.11)		12.93 (0.36)		1.76 (0.09)	

^a^ Adjusted for gender, marital status, education, food security, birthplace, PDQ, income to poverty ratio, age. ^b^ Adjusted for gender, marital status, education, food security, PDQ, income to poverty ratio, age. ^c^ Adjusted for education, birthplace, PDQ, age. ^d^ Adjusted for gender, education, employment, birthplace, PDQ, income to poverty ratio, age. Bolded numbers indicate *p*-value < 0.05.

**Table 5:** Adjusted means from multiple linear regression models of dinner HEI total score and components and cooking frequency groups among Non-Hispanic Black adults ≥ 19 years of age: NHANES 2007–2010 (observations = 2027; weighted population = 22,883,113).

	Total Dinner Vegetable ^a^ (Max Score 5)		Dinner Empty Calorie ^b^ (Max Score 20)		Dinner Seafood and Plant Protein ^c^ (Max Score 5)		Dinner Total Dairy ^d^ (Max Score 10)		Dinner Total Protein ^e^ (Max Score 5)	
	Adjusted Mean (SE)	*p* value	Adjusted Mean (SE)	*p* value	Adjusted mean (SE)	*p* value	Adjusted mean (SE)	*p* value	Adjusted Mean	*p* value
Low	2.66 (0.14)	0.072	**12.77 (0.57)**	**0.007**	0.56 (0.09)	0.083	**3.01 (0.32)**	**0.003**	**3.69 (0.15)**	**0.004**
Medium	2.71 (0.18)	0.096	13.56 (0.45)	0.094	**0.99 (0.07)**	**0.035**	**2.66 (0.16)**	**0.001**	4.02 (0.82)	0.323
High (reference group)	2.96 (0.12)		14.19 (0.46)		0.75 (0.09)		1.87 (0.13)		4.11 (0.09)	

^a^ Adjusted for marital status, education, employment, food security, PDQ, income to poverty ratio, age. ^b^ Adjusted for marital status, birthplace, age. ^c^ Adjusted for age. ^d^ Adjusted for birthplace and age. ^e^ Adjusted for birthplace and age. Bolded numbers indicate *p*-value < 0.05.

**Table 6:** Gender differences in meal preparer status and marital status among Non-Hispanic Black adults, ≥ 19 years of age NHANES 2007–2010. (main meal planner/ preparer weighted population = 25,255,835.32; marital status weighted population = 24,571,603.83).

	Female	Male	*p*-value
Observations	13,637	10,935	
Age	44.05	42.79	0.109
Main Meal Preparer			
Yes	77.2%	39.6%	**<0.001**
No	22.8%	60.4%	
Marital Status			
Married or living with a partner	37.5%	52.7%	**<0.001**
Previously or low married	62.5%	47.9%	

Weighted percentages are presented. Bolded numbers indicate *p* < 0.05.

## Supplementary Materials

The following are available online at https://www.mdpi.com/2072-6643/11/9/2057/s1, **Supplementary Figure S1 a-f**. Adjusted means for dietary quality by Total Daily and Dinner HEI scores by income categories: <130%, 131-185%, and > 185% income to poverty ratios.
