# Supplementary material for: Household Cooking Frequency of Dinner Among Non-Hispanic Black Adults is Associated with Income and Employment, Perceived Diet Quality and Varied Objective Diet Quality, HEI (Healthy Eating Index): NHANES Analysis 2007–2010
Source: Nutrients. 2019 Sep 2;11(9):2057. doi: 10.3390/nu11092057 (PMC6769568; doi:10.3390/nu11092057)
Supplement: Supplementary file 1 [file nutrients-11-02057-s001.pdf]

Supplement figure 1 a-f. Adjusted means for dietary quality by Total Daily and Dinner HEI scores by income categories: <130%, 131-185%, and > 185% income to poverty ratios. All models adjusted for gender, marital status, education, food security, birthplace, PDQ, and age. Reference group is high cook group. (observations = 2242, weighted population = 25,392,351 for daily; observations = 2027; weighted population = 22,883,113 for dinner). Bolded numbers indicate p-value <0.05.

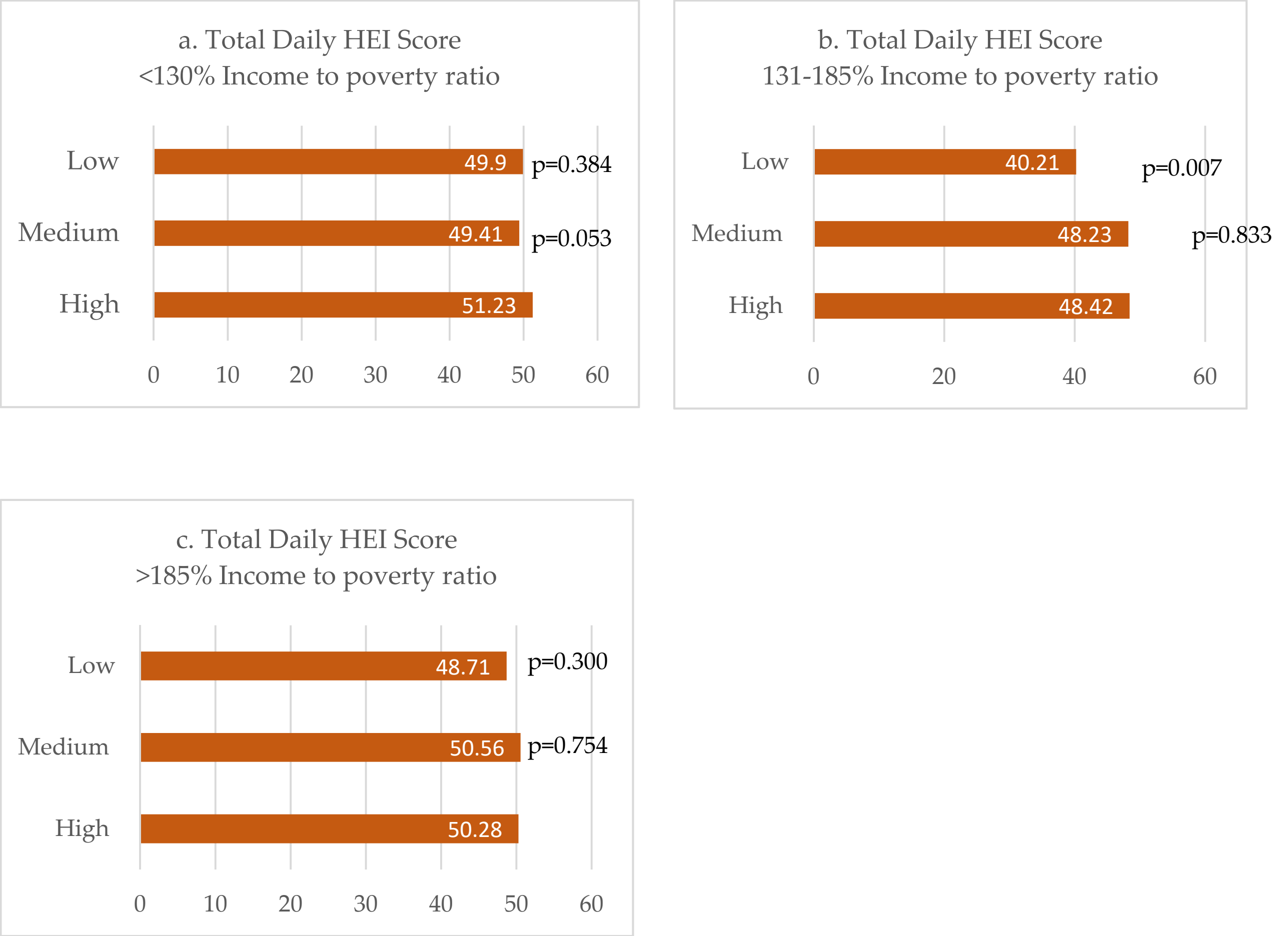

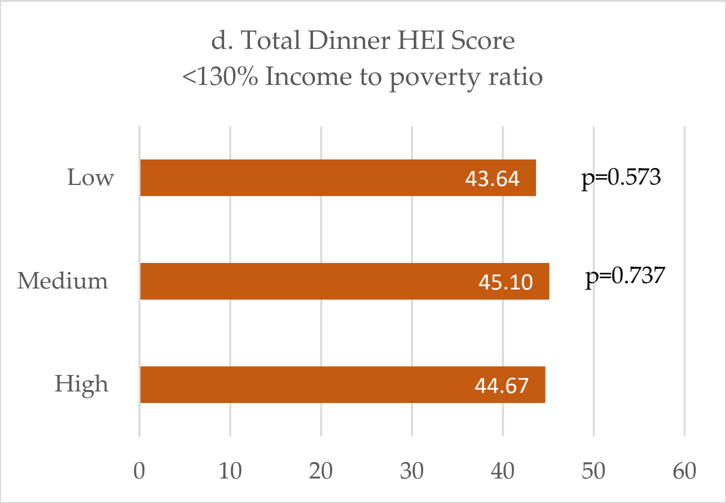
d. Total Dinner HEI Score
<130% Income to poverty ratio
Low
Medium
High
43.64
45.10
44.67
p=0.573
p=0.737
0
10
20
30
40
50
60

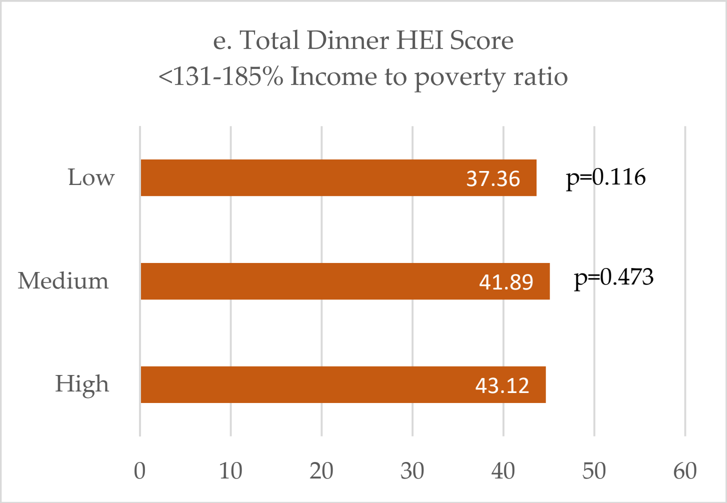
e. Total Dinner HEI Score
<131-185% Income to poverty ratio
Low
Medium
High
37.36
41.89
43.12
p=0.116
p=0.473
0
10
20
30
40
50
60

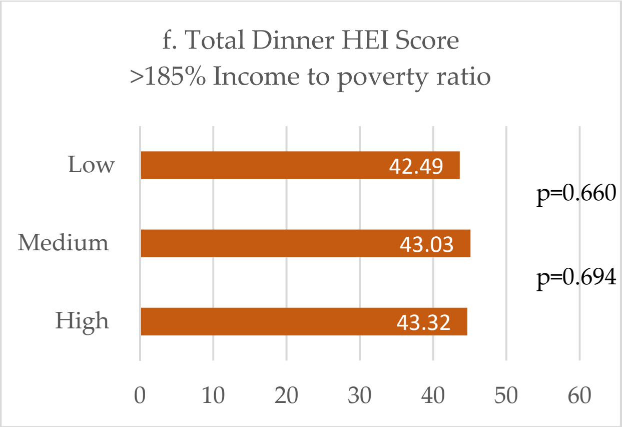
f. Total Dinner HEI Score
>185% Income to poverty ratio
Low
Medium
High
42.49
43.03
43.32
p=0.660
p=0.694
0
10
20
30
40
50
60
